# Cyclic AMP signalling pathways in the regulation of uterine relaxation

**DOI:** 10.1186/1471-2393-7-S1-S10

**Published:** 2007-06-01

**Authors:** Wei Yuan, Andrés López Bernal

**Affiliations:** 1 University of Bristol, Clinical Science at South Bristol (Obstetrics and Gynaecology), St Michael's Hospital and Dorothy Hodgkin Building, Whitson Street, Bristol, BS1 3NY, UK

## Abstract

Studying the mechanism(s) of uterine relaxation is important and will be helpful in the prevention of obstetric difficulties such as preterm labour, which remains a major cause of perinatal mortality and morbidity. Multiple signalling pathways regulate the balance between maintaining relative uterine quiescence during gestation, and the transition to the contractile state at the onset of parturition. Elevation of intracellular cyclic AMP promotes myometrial relaxation, and thus quiescence, via effects on multiple intracellular targets including calcium channels, potassium channels and myosin light chain kinase. A complete understanding of cAMP regulatory pathways (synthesis and hydrolysis) would assist in the development of better tocolytics to delay or inhibit preterm labour. Here we review the enzymes involved in cAMP homoeostasis (adenylyl cyclases and phosphodiesterases) and possible myometrial substrates for the cAMP dependent protein kinase. We must emphasise the need to identify novel pharmacological targets in human pregnant myometrium to achieve safe and selective uterine relaxation when this is indicated in preterm labour or other obstetric complications.

## Background

Preterm labour is a major reproductive health problem due to a high incidence of severe short- and long-term infant morbidity. In developed countries, social and medical advances have improved the survival rate of the preterm neonate; however, the incidence of preterm birth and related morbidity remain a serious challenge and the physiopathological mechanisms of preterm birth are still a mystery. The use of tocolytic drugs, including those that operate through cyclic AMP such as beta-mimetics, to inhibit uterine contractility in preterm labour is controversial because there is no evidence that currently available drugs improve long term neonatal outcome. Moreover, some tocolytics can cause serious side effects such as tachycardia, hypertension and pulmonary oedema. The potential of drugs of high uterine selectivity, e.g. oxytocin receptor antagonists, for the management of preterm labour is encouraging and further clinical trials are being undertaken. However, there is a need to identify novel pharmacological targets in myometrium to provoke safe and selective uterine relaxation when this is clinically indicated [1].

It is not known whether the cause of preterm labour is the premature loss of uterine quiescence (e.g. removal of inhibitory factors), or the induction of uterine contractility (e.g. release of stimulatory mediators) or a combination of both [2]. The second messenger cyclic adenosine monophosphate (cAMP) is known to promote the relaxation of smooth muscle, and is likely to be implicated in the maintenance of uterine quiescence [2]. Hence, the study of myometrial cAMP regulatory pathways will help to understand the mechanism of labour and highlight possible targets for the development of more specific and effective tocolytics for preterm labour.

## Cyclic AMP signalling pathways

**Figure 1:**
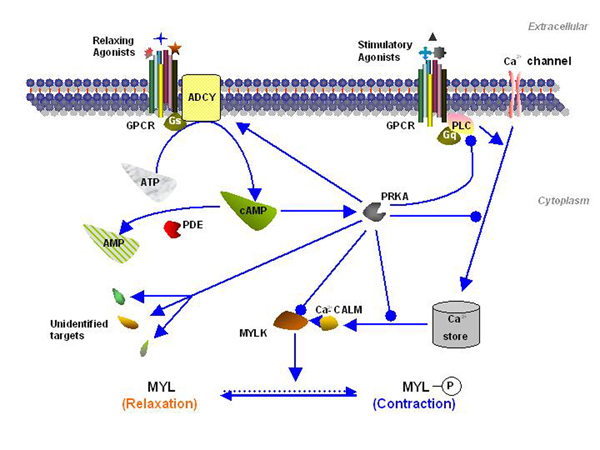
**Cyclic AMP pathways in myometrial tissue**. Activation of membrane receptors (GPCR) coupled to Gs activates adenylyl cyclase (ADCY) which converts ATP to cAMP. The levels of cAMP are tightly regulated by phosphodiesterases (PDE), especially PDE4 isoforms. It is thought that cAMP induces uterine relaxation via activation of a specific protein kinase (PRKA) which phosphorylates and inhibits myosin light chain kinase (MYLK). PRKA may also oppose the effect of stimulatory receptors which operate through the phospholipase C (PLC)/calcium pathway. However the precise targets for PRKA phosphorylation in human myometrium are under investigation ( stimulation,  inhibition)

Cyclic AMP is a diffusible intracellular second messenger, which influences many physiological events, by transducing hormone and small molecule effects into activation of protein kinases, modulating calcium transport and regulating gene activation. Its function in the relaxation of uterine and other types of smooth muscle is believed to be via inhibition of calcium mobilization and the contractile apparatus [3], through the activation of cAMP-dependent protein kinase (PRKA), which phosphorylates target proteins such as myosin light chain kinase (MYLK) [4] and phospholipase C (PLC) [5] (Table 1 and Figure 1). However the identification of PRKA substrates in human myometrium is a challenging area of research and more information is required before the mechanism of cyclic nucleotide-induced relaxation is understood.

The synthesis and catabolism of cAMP has been studied in many systems. Adenylyl cyclases (ADCY) catalyse the conversion of ATP to cAMP in response to the actions of hormones and drugs acting upon cell surface G protein coupled receptors. The cAMP formed is hydrolysed by a family of cyclic nucleotide phosphodiesterases (PDE). In myometrium ADCY activity is induced by endogenous agonists (catecholamines, prostaglandins, etc) operating through protein receptors e.g. ADRB2 (β2-adrenoceptor), PTGER2 (prostaglandin E2 receptor) coupled to Gs [6]. Activated ADCY removes one pyrophosphate molecule to convert ATP to cAMP and releases it into the cytoplasm. Cyclic AMP activates PRKA by binding to its catalytic subnits. Spatial and temporal changes in cAMP levels can be translated into compartmentalised responses by PRKA anchoring proteins which also bind other protein kinases and PDE isoforms to regulate a variety of signalling activities including feedback phosphorylation of ADCY [7]. The concentration of cAMP is reduced rapidly by PDE hydrolysis. Regulation of PDEs, especially PDE4, the largest myometrial PDE family, is an attractive area of study because of its potential as a new target for tocolysis. Long forms of PDE4 are involved in fast cAMP signalling loops through PRKA activation at conserved phosphorylation domains, while short forms of PDE4 are associated with long term responses promoted by cAMP such as gene transcription. Moreover, prostaglandin E2 up-regulates PDE activity by inducing the synthesis of PDE4 short isoforms [8].

## Adenylyl cyclases and their regulators

Is there a specific myometrial cAMP generation system? This question is important because it would provide a target for pharmacological intervention. ADCY are a family of disparate enzymes directly involved in cAMP synthesis. The catalytic core of mammalian ADCY consists of a pseudosymmetric heterodimer composed of two highly conserved cytosolic regions, namely C_1a _and C_2a_, which can bind Gs and ATP. All isoforms of ADCY are activated by the α subunit of Gs (Gαs) and most are directly activated by the diterpene forskolin, which is a useful tool for functional studies [9,10]. The C_1a _and C_2a _regions alone are sufficient for cAMP generation, and linkage of portions of these cytosolic domains yields a soluble form of ADCY, which is fully activated by both Gαs and forskolin [11,12].

So far, nine mammalian membrane-bound isoforms of ADCY have been identified, and allocated into one of four subgroups, according to their modes of regulation [9] (table 2). All isoforms are activated by Gαs binding primarily to a hydrophobic, negatively charged pocket on the C_2a _domain [10]. Since the site of interaction of Gαs with ADCY is distal to the catalytic site, it has been suggested that the likely mechanism for Gαs activation involves a modulation of the structure of the active site by the induction of a conformational change. All ADCYs, except ADCY 9, which lacks the required serine and leucine residues, can be activated by forskolin, which binds in a narrow hydrophobic crevice between the C_1a _and C_2a _domains [13]. Activation of ADCYs by forskolin occurs even in the absence of a functional G protein, and is, therefore, a consequence of a direct action on the catalytic subunit of the enzyme [14].

Responses of ADCYs to other modulators can be very different. For example, Group 1 cyclases (ADCY1, ADCY3, ADCY8) are stimulated by calcium/calmodulin (Ca^2+^-CALM) and by PRKA but are inhibited by the α subunits of proteins of the Gαi/o family and also by G protein βγ subunits (G_βγ_). In contrast, Group 2 isoforms (ADCY2, ADCY4, and ADCY7) are activated by G_βγ _and insensitive to Ca^2+^-CALM [15]. Group 3 isoforms (ADCY5 and ADCY6) are similar to Group 1, in that they are inhibited by G_αi/o _and G_βγ_, but they are also inhibited by the pertussis toxin-insensitive inhibitory G-protein Gαz, by low concentrations of calcium, and by PRKA phosphorylation at Ser-674 [16]. ADCY9 belongs in a separate group because it is insensitive to either calcium or G_αi/o_/G_βγ_, but is inhibited by the phosphatase calcineurin [17]. The effects of protein kinase C (PRKC) on ADCY are very complex and cut across groups. For instance PRKC phosphorylation enhances the activities of ADCY2 and ADCY7 (Group 2) as well as ADCY5 (Group 3). On the other hand phosphorylation by PRKC reduces the activities of ADCY4 (Group2) and ADCY6 (Group 3) [9,15]. In pregnancy there is upregulation of group 2 and group 3 isoforms in myometrium [18], but the question whether there are dominant ADCY isoforms coupled to specific myometrial receptors or whether there is promiscuity in the coupling of GPCR/Gαs complexes to several ADCY isoforms remains unanswered. The development of sensitive fluorescent tags to study molecular proximity in real time will help answer this question in myometrial cells.

## Phosphodiesterases and their regulators

The phosphodiesterases (PDE) multi-gene family of enzymes catalyze the hydrolysis of 3',5'-cyclic nucleotides, and so occupy a key position in modulating intracellular cyclic nucleotide levels [19]. In myometrial tissue, the activity of PDE enzymes is significantly inhibited during pregnancy [20], probably as a consequence of the high progesterone levels [21], suggesting that cAMP-PDE inhibition may be part of the mechanism for myometrial relaxation and pregnancy maintenance.

At present, more than 14 PDE genes have been cloned in mammals and 21 gene products have been identified. PDE isoforms are grouped in 11 distinct but related families (PDE1 to PDE11), according to sequence homology, substrate specificity, inhibitor sensitivity and function. Within these families alternative splicing or mRNA transcription initiation result in additional diversity producing around 100 mRNA products, but it is not clear how many of these are actually translated into different proteins [19,22]. The protein isoforms all share a common structural pattern: a C-terminus whose function is unclear; a conserved catalytic core, which determines substrate specificity and inhibitor sensitivity; and a divergent N-terminus responsible for the enzyme's targeting [19,22].

PDE subfamilies are differentially expressed in human tissues, and only PDE1-5 subtypes have been detected in human term myometrium [23]. PDE1 subtypes are of mixed substrate specificity for cyclic nucleotides (they tend to hydrolyse either cAMP or cGMP) and contain auto-inhibitory substrate-like sequences; such inhibition is relieved by Ca^2+^/CALM [24]. PDE1 isoforms are clearly regulated by PRKA and are involved in cell proliferation [25].

PDE2 and PDE5 have similar structures as both of them contain allosteric cyclic nucleotide binding sites for cGMP. The binding of cGMP to PDE2 stimulates cAMP hydrolysis to mediate cross-talk between cGMP and cAMP pathways [26,27]. PDE5 expression is inhibited by hCG in human myometrium [28], and some evidence suggested that PDE5 may play a role in myometrial cell proliferation and cGMP dependent potassium channel regulation. High doses of sildenalfil, a specific PDE5 inhibitor, relax human pregnant myometrium in vitro, but this effect is unlikely to be of tocolytic value [29].

PDE3 has two isoforms PDE3A and PDE3B, which contain a conserved catalytic site for both cAMP and cGMP binding [30]. Functional studies suggest that PDE3 is involved in vascular smooth muscle contractions, heart function, platelet aggregation, oocyte maturation and cell metabolism [31].

PDE4 is the largest PDE subfamily, consisting two classes of isoforms, long or short PDE4, depending upon the presence or absence of upstream conserved regions. Both classes of PDE4 have a much higher affinity for cAMP than for cGMP [32]. In human myometrial cell, the major source (up to 75%) of PDE activity is provided by PDE4 isoforms and membrane targeting can influence the maximal activity of the enzyme and its sensitivity [23,33]. Early functional studies of PDE4 showed that specific inhibition with rolipram leads to inhibition of the spontaneous contraction of myometrial strips [34,35]. However, rolipram has significant side effects, particularly nausea and vomiting. Cilomilast, originally designed as a novel therapy for asthma with fewer side effects, produces concentration-dependent inhibition of the spontaneous contractions of term myometrium [36]. PDE4 selective inhibitors reduce the incidence of inflammation-induced preterm delivery in mice [37]. A better understanding of the regulation of PDE4 isoforms in pregnant human myometrium may be a useful approach to develop tocolytics of high uterine selectivity [33].

## Conclusion

The role of cAMP as an important mediator in the regulation of myometrial function is beyond dispute. Much is known about the pathways for cAMP synthesis and hydrolysis. However, the mechanisms of action of cAMP and its associated kinase PRKA in myometrial cells are not fully understood. Several candidate PRKA substrates have been proposed based on studies in other types of smooth muscle. More research into the identification of physiological PRKA substrates in pregnant human myometrium and into their regulation by phosphorylation will clarify the role of cAMP in pregnancy maintenance, and provide novel targets for uterine-selective therapeutic intervention.

## List of abbreviations used

ADCY, adenylyl cyclases; ADRB2, β2-adrenoceptor; CALM, calmodulin; cAMP, cyclic AMP (cyclic adenosine monophosphate); GPCR, G protein-coupled receptor; MYL, myosin light chain kinase; PDE, cyclic nucleotide phosphodiesterase; PLC, phospholipase C; PRKA, cAMP-dependent protein kinase; PRKC, protein kinase C; PTGER2, prostaglandin E2 receptor.

## Competing interests

The authors declare that they have no competing interests.

## Authors' contributions

WY drafted the manuscript. ALB conceived the study, and helped to draft the manuscript. All authors read and approved the final manuscript.

## Figures and Tables

**Table 1:** Potential protein kinase A substrates involved in the regulation of human uterine relaxation

**Physiological roles (see Shabb (2001) [38])**	**Protein substrate (HUGO nomenclature)**	**References**
Autophosphorylation	cAMP dependant protein kinase regulatory subunit type IIα (PRKAR2A)	Zakhary *et al. *(2000) [39]
cAMP signalling	β-2 adrenoceptor (ADRB2)	Daaka Y *et al. *(1997) [40]; Iyer *et al. *(2006) [41]
	G protein coupled receptor kinase-2 (ADRBK1)	Houslay & Baillie (2006) [42]
	Phosphodiesterase 4 (PDE4)	Murthy *et al. *(2002) [43]
Phosphoinositide and calcium signalling	InsP3 Type I receptor (ITPR1)	Straub *et al. *(2004) [44]
	Phospholipase-C β3 (PLCB3)	Yue *et al. *(1998) [45]
	Phospholipase-C γ1 (PLCG1)	Park *et al. *(1992) [46]
	ATPase 2 (ATP2)	Tribe *et al. *(2000) [47]
	Regulators of G-protein signalling (RGS)	Suarez *et al. *(2003) [48]
	Thromboxane A2 receptor (TBXA1R)	Walsh *et al. *(2000) [49]
Rho signalling	RhoA small GTP binding protein (RHOA)	Murthy *et al. *(2003) [50]
	Gα13	Manganello *et al. *(2003) [51]
Smooth muscle contraction	Myosin light chain kinase (MYLK)	Hirano *et al. *(2004) [52]
	Myosin binding subunit of myosin phosphatase (PPP1R12A)	Wooldridge *et al. *(2004) [53]

**Table 2:** Adenylyl cyclase (ADCY) isoforms

**Sub-family**	**Mode of regulation**	**Member**	**Chromosomal localization**	**Referen ces**
1	Activated by calcium, Gαs, PRKA inhibited by Gαi/o and Gβγ	ADCY1	7p12	Villacres *et al. *(1993) [54]
		ADCY3	5p15	Diel *et al. *(2006) [55]
		ADCY8	2p22-24	Steiner *et al. *(2006) [56]
2	Activated by Gαs and Gβγ	ADCY2	14q11.2	Nguyen & Watts (2006) [57]
		ADCY4	3q13.2-q21	Wang & Burns (2006) [58]
		ADCY7	12q12-13	Guzman *et al. *(2005) [59]
3	Activated by Gαs, Inhibited by Gαi/o, Gβγ calcium, PRKA	ADCY5	16q12-13	Iwami *et al. *(1995) [60]
		ADCY6	8q24	Chen *et al. *(1997) [61]
4	Activated by Gαs	ADCY9	16p13	Hacker *et al. *(1998) [62]
